# Eco‐Friendly and Self‐Sanitizing Microporous Cellulose Sponge (MCS)‐Based Cooling Media for Mitigating Microbial Cross‐Contamination in the Food Cold Chain

**DOI:** 10.1002/advs.202309753

**Published:** 2024-03-28

**Authors:** Yijun Liu, Boyang Xu, Yingxin Li, Siew‐Young Quek, Kang Huang

**Affiliations:** ^1^ School of Chemical Sciences The University of Auckland Auckland 1142 New Zealand; ^2^ Department of Biological Systems Engineering Washington State University Pullman WA 99164 USA

**Keywords:** alternative ice cubes, cellulose sponge, cold supply chain, photosensitizer, self‐sanitizing activity, sustainable and reusable cooling media

## Abstract

Maintaining precise temperature control is vital for cold chain food transport, as temperature fluctuations can cause significant food safety and quality issues. During transport, ice that melts can promote the growth of microbes and their spread, resulting in microbial cross‐contamination. This study developed sustainable, non‐melting, self‐sanitizing “ice cubes” using food grade compositions including microporous cellulose sponges (MCS) and photosensitizers, aimed at enhancing temperature regulation and minimizing microbial contamination in the cold chain. Upon absorbing water, the MCS matched traditional ice in cooling efficiency and heat absorption and exhibit remarkable mechanical and thermal durability, withstanding multiple freeze‐thaw cycles and compressive stresses. The cationic MCS combined with erythrosine B demonstrated strong self‐sanitizing capabilities, effectively reducing microbial cross‐contamination in food models. Additionally, the release rates of photosensitizers from the MCS can be modulated by altering environmental ionic strength. This research offers viable solutions to address microbial cross‐contamination challenges in current cold chain systems.

## Graphical abstract

This study introduces sustainable, non‐melting, self‐sanitizing “ice cubes” made from microporous cellulose sponges (MCS) and food‐grade photosensitizers, offering a novel solution to enhance cold chain systems. These “ice cubes” match traditional water‐based ice in cooling efficiency, withstand multiple freeze‐thaw cycles, and effectively reduce microbial cross‐contamination, presenting a significant advancement in temperature regulation and food safety.

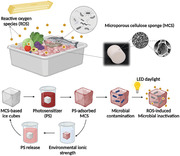

## 1 Introduction

Accurate temperature control is essential for the cold chain transport of food products, with any temperature fluctuation potentially leading to significant food safety and quality issues. The consensus in the food industry identifies temperature mishandling as a primary cause of food spoilage and a factor in the degradation of quality, often resulting in offensive odors^[^ ^1^ ^]^ and potentially harmful by‐products that pose health risks.^[^ ^2^, ^3^ ^]^ Approximately 50% of losses occur due to suboptimal conditions during the pre‐cooling, transportation, and storage steps.^[^ ^4^ ^]^ The current widespread use of ice as a coolant carries the risk of ice melting and microbial cross‐contamination. Ice melting during transport can foster pathogenic microbial growth and facilitate their spread, leading to microbial cross‐contamination.^[^ ^5^, ^6^, ^7^ ^]^ Statistics indicate that cross‐contamination during transport, especially over long distances, accounts for 39% of foodborne illnesses, with temperature instability contributing to half of these cases.^[^ ^8^ ^]^ Additionally, ice contamination has been implicated in gastrointestinal disorders and nosocomial infections.^[^ ^9^ ^]^ These statistics highlight the critical need for more reliable and safer cooling alternatives in transporting perishable foods.

In response to the significant challenges posed by traditional cooling media in the food cold chain, the last two decades have seen the development of various alternatives aimed at mitigating microbial cross‐contamination risks. The initial efforts involved the addition of chlorine to the ice to inhibit microbial activity;^[^ ^10^, ^11^ ^]^ however, this led to the creation of disinfection by‐products (DBPs) such as trihalomethanes when chlorine interacted with organic matter.^[^ ^12^, ^13^, ^14^ ^]^ These DBPs have been linked to health concerns, prompting certain regions, such as the European Union, to prohibit such practices.^[^ ^13^ ^]^ In addition to chlorine, other chemical antimicrobial agents such as essential oils and acidic electrolyzed water were added into ice cubes,^[^ ^9^, ^15^, ^16^ ^]^ while effective in inactivating microbes, their drawbacks include potential toxicity and impact on the sensory attributes of food. Subsequently, innovations such as antimicrobial ice and reusable ice packs have been introduced. Despite these advancements, their widespread adoption has been limited due to issues such as altered taste from antimicrobial agents and safety considerations regarding the materials used in ice packs.^[^ ^1^, ^17^ ^]^ Silicone or plastic ice cubes offer a cost‐effective solution, but concerns over potential leakage and the breakdown of polymeric materials including microplastic issues pose health risks. Stone‐based alternatives provide a safer option but fall short on cooling efficacy and transport convenience, rendering them suboptimal for large‐scale cold chain logistics. Stainless‐steel ice cubes, primarily used to chill drinks, carry a prohibitive cost for broader cooling applications. A noteworthy development is the use of gelatin‐based hydrogels as alternative ice cubes;^[^ ^18^, ^19^, ^20^ ^]^ A recent study successfully incorporated menadione sodium bisulfite into gelatin hydrogels, enabling light‐activated antimicrobial properties against bacteria, fungi, and yeast.^[^ ^21^ ^]^ However, their physical properties, including mechanical and thermal stability, deteriorate after multiple freeze‐thaw cycles. Moreover, derived from animal collagen, gelatin‐based materials may pose allergenicity and health risks for some individuals and conflict with the dietary restrictions of certain religious groups, thus limiting their widespread acceptance. In addition, there are concerns regarding disease transmission through protein‐based materials, which could compromise the effectiveness of disinfection processes due to the presence of high organic content, whether relying on the application of chlorine‐based sanitizers or the inherent antimicrobial properties of materials.^[^ ^19^ ^]^ Consideration has also been given to ice packs with polymer casings. Though innovative, they are found to reduce energy efficiency in thermal and cold energy storage and raise potential environmental concerns due to excessive plastic use.^[^ ^22^, ^23^ ^]^ Therefore, there is an unmet need to develop new solutions that can overcome current limitations by providing safe, efficient, and cost‐effective temperature control without compromising food safety or sensory qualities.

Cellulose, as the most abundant biopolymer on Earth, offers several advantages over petroleum and inorganic‐based materials. Its widespread availability, ease of fabrication, biodegradability, and affordability make it an attractive alternative. Leveraging cellulose as a foundational material shows excellent promise for cost‐effectiveness in developing new products. The molecular structure of cellulose is inherently rich in hydrophilic functional groups, such as hydroxyl and carboxyl groups, positioning it as an excellent candidate for creating highly adsorbent systems. The practical applications of cellulose porous networks such as aerogels, sponges, and hydrogels are diverse, including personal hygiene products such as diapers and sanitary pads, and extending to medical applications such as drug delivery systems.^[^ ^24^ ^]^ Furthermore, these cellulose porous materials have demonstrated efficacy in environmental applications, such as adsorbing heavy metals and dyes from wastewater,^[^ ^25^, ^26^, ^27^ ^]^ demonstrating the versatility and utility of cellulose in various sectors. Therefore, leveraging the advantages of cellulose materials for food‐related applications holds significant promise for the food industry.

**Figure 1:**
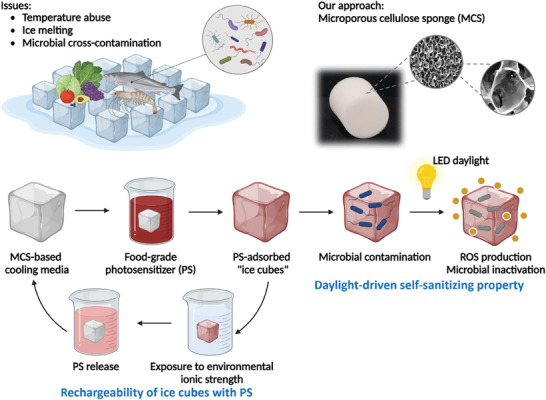
Schematic diagram of the current issues associated with water‐based ice cubes and overall designs of our approach using MCS combined with photosensitizers.

In this study, we engineered eco‐friendly, non‐melting, self‐sanitizing “ice cubes” using microporous cellulose sponges (MCS) and photosensitizers, designed to enhance temperature control and reduce microbial contamination within the cold chain. By cross‐linking cellulose with citric acid, we developed a food‐grade, biodegradable MCS that forms a mesoporous polymer network, conferring the material with exceptional mechanical properties and a robust capacity for photosensitizer adsorption. These innovative MCS‐based “ice cubes” offer several key advantages (**Figure** **1**): a) the bactericidal properties of MCS, when combined with adsorbed photosensitizers, can combat a spectrum of pathogenic microbes, thus mitigating the risk of microbial cross‐contamination during cold chain transport and storage; b) the proposed MCS is reusable, maintaining its mechanical and thermal integrity even after numerous freeze‐thaw cycles, and its ability to adsorb and release photosensitizers can be regulated by environmental ionic strength, facilitating the recharging process of the self‐sanitizing “ice cubes”; c) the MCS is highly compressible and regain their shape upon the release of pressure, enhancing space efficiency in storage and transport and subsequently lowering costs; and d) this sustainable compositions offer a scalable and economical alternative to conventional methods. The findings of this research have the potential to overcome significant scientific challenges faced by the existing cold chain infrastructure.

## 2 Results and Discussion

### 2.1 Design and Preparation of MCS

In this study, we aimed to develop a MCS‐based cooling medium that is sustainable, self‐cleaning, and reusable for applications in the food cold chain. The ideal MCS would possess the following key characteristics: a) high water adsorption capacity to maximize the quantity of water that can be frozen; b) strong mechanical properties to withstand numerous freeze‐thaw and dehydration‐rehydration cycles; c) regulated adsorption and controlled release of photosensitizer (PS) molecules upon soaking to confer rechargeable self‐sanitizing capabilities. To achieve these characteristics, the proposed MCS was synthesized through the cross‐linking between sodium carboxymethyl cellulose (NaCMC) and quaternized hydroxyethyl cellulose ethoxylate (QHEC) using citric acid (CA). This combination was chosen for its simplicity and safety for food applications compared with other strategies utilizing nanocellulose that are typically difficult to disperse or necessitating hazardous cross‐linking approaches involving epoxides or ionizing radiations.^[^ ^28^, ^29^, ^30^, ^31^ ^]^ The creation of 3D cellulose porous structures during the cross‐linking provides the desired properties for effective use in the food cold chain.

**Figure 2:**
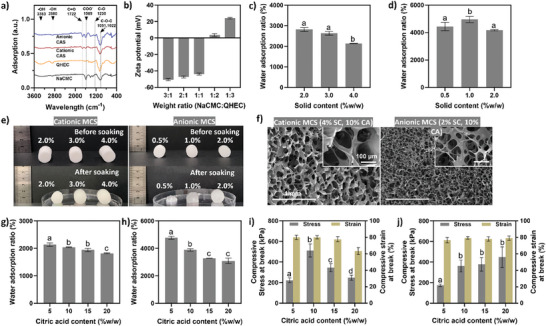
Comprehensive analysis of the synthesized MCS: a) ATR‐FTIR spectra of cellulose materials and synthesized MCS; b) zeta potential measurements of pre‐gel mixtures with varying NaCMC to QHEC ratios; comparative water adsorption ratios of c) cationic and d) anionic MCS at various polymer solid concentrations; e) visual comparison of MCS pre‐ and post‐soaking in water. f) SEM images illustrating the microstructural differences between cationic MCS and anionic MCS; water adsorption behavior of g) cationic MCS and h) anionic MCS in relation to varying citric acid levels; and compressive strength of i) cationic MCS and j) anionic MCS with differing citric acid concentrations. Data are expressed as mean ± SD of at least three replicates. Means with different letters on the bars (a – d) represent significant difference (*p* < 0.05).

The successful chemical linkage between cellulose materials and CA was confirmed using Attenuated Total Reflection‐Fourier Transform Infrared (ATR‐FTIR) spectroscopy, as shown in **Figure** **2**a. The spectra for both NaCMC and QHEC samples revealed characteristic carboxylate (COO‐) bands at 1592, 1413, and 1319 cm^−1^, as well as ether (C─O─C) bands at 1052 and 1022 cm^−1^.^[^ ^32^ ^]^ Cross‐linking of CA with cellulose materials, occurs via molecular dehydration of the acid, followed by esterification. This reaction resulted in a new peak at 1722 cm^−1^, indicative of the ester groups and carbonyl groups from CA. Moreover, a notable shift in the hydroxyl (OH) peak intensity ≈3400–3200 cm^−1^ was evident, particularly by the absorbance at 3383 and 2880 cm^−1^, corresponding to the stretching vibrations of OH groups involved in hydrogen bonding.^[^ ^32^ ^]^ The increased vibration at 1230 cm^−1^ in the cross‐linked MCS points to the C─O stretching of the newly formed ester groups.^[^ ^33^ ^]^ Since electrostatic interactions play a decisive role in adsorbing charged species from aqueous environments,^[^ ^34^ ^]^ we proposed to manipulate the surface charge of MCS by varying the ratio of positively and negatively charged components in the formula. Figure 2b presents the net surface charges of the MCS synthesized with varying NaCMC and QHEC weight proportions. It was found that MCS with 1:2 and 1:3 weight ratios exhibited positive charges, whereas those with 3:1, 2:1, and 1:1 weight ratios displayed negative surface charges. Notably, a MCS with equal weights of NaCMC and QHEC demonstrated a particularly negative charge (−43.84 ± 1.27 mV), reflecting the high negative charge intrinsic to NaCMC molecules.^[^ ^35^ ^]^ Results in Figures S1 and S2 (Supporting Information), demonstrate that the adsorption of cationic photosensitizers, such as methylene blue (MB), is enhanced by higher concentrations of NaCMC in the MCS formulation. Conversely, an increased proportion of QHEC facilitates the rapid uptake of anionic photosensitizer molecules such as erythrosine B (EB). To optimize the adsorption of ionic photosensitizers, we selected NaCMC to QHEC ratios of 3:1 and 1:3 for synthesizing anionic and cationic MCS, respectively, for subsequent experiments. Notably, such strategic selection of polymer ratios was crucial in tailoring the surface charge and functionality of MCS to meet other desired application requirements.

To optimize the water adsorption and mechanical strength of the synthesized MCS, the optimal concentrations of polymer solids and cross‐linker (CA) were determined. The cationic MCS were formulated using a 1:3 NaCMC to QHEC ratio, with polymer solid concentrations of 2.0%, 3.0%, and 4.0% by weight. Conversely, the anionic MCS, formulated with a 3:1 ratio of NaCMC to QHEC, were prepared with 0.5%, 1.0%, and 2.0% polymer solids. Across all formulations, the CA content was consistently maintained at 10% w/w relative to the polymer solids. The water adsorption and structural integrity of MCS after soaking are key indicators for determining the optimal polymer solid content. All formulations exhibited high water adsorption, with adsorption ratios exceeding 2000%, as shown in Figure 2c,d. Notably, the water adsorption ratios for cationic MCS were lower than those for anionic MCS, likely due to differences in cross‐linking degrees. In cationic MCS, a higher polymer solid content led to a denser cross‐linking network, reducing adsorption capacity due to limited space within the sponge network. This trend was evident in Figure 2e, which demonstrated the structural integrity of the MCS after soaking. Specifically, the cationic MCS with a 4.0% solid content preserved its structure post‐soaking overnight, whereas those with only a 2.0% solid content suffered damage under the same conditions. Anionic MCS showed a similar pattern, with lower solid contents of 0.5% and 1.0% resulting in shape loss. Based on these observations, we selected a polymer solid content of 4.0% w/w for cationic MCS and 2.0% w/w for anionic MCS, as these concentrations provided the optimal balance between water adsorption capacity and structural integrity.

Citric acid (CA) content is another determinant factor that influences the water adsorption behavior and mechanical properties of MCS, as it is directly related to the density of network cross‐linking. For both cationic and anionic MCS, an increase in CA content was observed to reduce the water adsorption ratios, attributed to the formation of a tighter cross‐linked network. However, as shown in Figure 2g,h, all the MCS maintained a adsorption ratio greater than 1800%. Importantly, both cationic and anionic MCS demonstrated no significant volume expansion upon immersion in water (Figure S3, Supporting Information). This behavior is attributed to its unique microscopic structure and the density of cross‐linking within the material, which prevents the typical volumetric expansion commonly observed in other hydrogel systems.^[^ ^36^, ^37^, ^38^ ^]^ Moreover, varying the CA content significantly affected the mechanical properties of the MCS. Results in Figure 2i indicate that the cationic MCS with a CA concentration of 10% w/w exhibited higher compressive stress at break and strain percentage than other formulations. Likewise, results in Figure 2j demonstrate that anionic MCS with a 10% w/w CA concentration were more resilient under compressive stress than those with a 5% w/w concentration. However, increasing the CA content beyond 10% w/w up to 20% w/w did not further enhance the mechanical properties of the MCS. Based on these findings, a CA content of 10% w/w was deemed optimal for both cationic and anionic MCS and was thus chosen for subsequent studies. This concentration of CA provided an ideal balance between water adsorption efficiency and mechanical robustness, aligning well with our objectives of creating effective and durable MCS for practical applications.

In summary, the method for creating porous MCS encompasses two principal steps: pore formation through lyophilization and cross‐linking using food‐grade citric acid, setting it apart from traditional cellulose sponge manufacturing techniques. Conventional methods rely on the cellulose xanthate process and a hydrothermal procedure, often involving the use of chemicals such as carbon disulfide^[^ ^39^ ^]^ or sodium borohydride^[^ ^40^ ^]^ to create porous networks. These substances, known for their potential to release toxic gases, are not preferred for applications in the food industry due to their high toxicity. In contrast, our approach eliminates the need for pore‐forming salt crystals or gas‐generating chemicals. The microscopic images in Figure 2f demonstrate that the cavities in the MCS were pre‐defined in the lyophilization and subsequent cross‐linking processes, where the cavities were enclosed by highly cross‐linked cellulose molecules that merely expand its volume while soaked in water. The lyophilization and cross‐linking processes are crucial for preserving the porous structure formed by ice crystals, essential for maintaining the dimensional stability of cellulose sponges under wet conditions. This approach enhances the safety profile of the MCS for food‐related applications without compromising on the product's utility.

### 2.2 Dehydration and Rehydration Cycles

**Figure 3:**
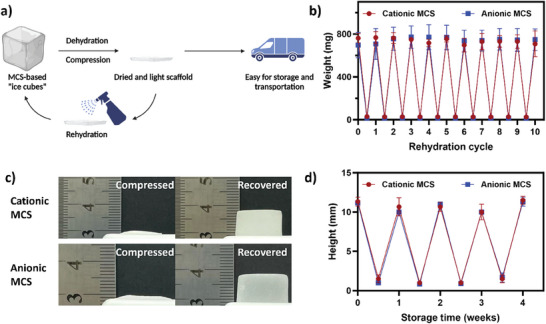
Assessment of dehydration and rehydration cycles: a) Schematic illustration of the dehydration and rehydration cycles experienced by the MCS; b) weight changes of the MCS over ten cycles of dehydration and rehydration; c) representative photographs of the MCS subjected to compression, vacuum‐pack, and rehydration; and d) Height changes of the MCS after being rehydrated from a vacuum‐packed state over a period of four weeks. Data are expressed as mean ± SD of at least three replicates.

As potential reusable cooling materials, the ability of MCS to undergo multiple dehydration and rehydration cycles is critical for their recycling efficiency, longevity, and practicality in applications, storage, and transportation. In this assay, the MCS were dehydrated at 30 °C for 24 h and then rehydrated in water for an equivalent period. **Figure** **3**b shows that after 10 cycles, the MCS maintained its water adsorption capacity (*p* > 0.05), affirming the durability for both cationic and anionic types. Additionally, the MCS underwent compression and vacuum‐sealing for up to four weeks to simulate real‐world storage and transport conditions. After each week, the MCS were unsealed and rehydrated, with its recovery assessed by measuring their height. As shown in Figure 3c, post‐compression, the MCS, initially compressed to less than 1 mm, successfully regained its original height of ≈11 mm upon rehydration. The findings presented in Figure 3d show no significant changes in height after four weeks of vacuum storage, indicating the exceptional mechanical stability of the material against mechanical stresses and the dehydration process. This lightweight and stable material offers the potential for significant reductions in transportation and storage costs compared to traditional water‐based ice cubes.

### 2.3 Controlled Adsorption and Release Kinetics of Photosensitizers

**Figure 4:**
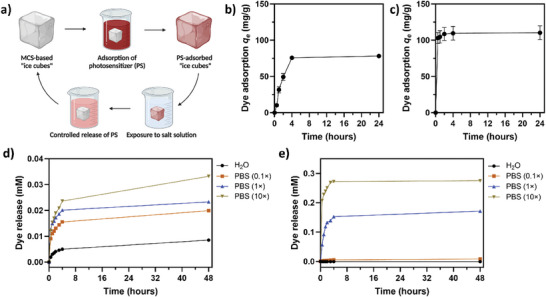
Adsorption and controlled release of photosensitizers by the synthesized MCS: a) schematic diagram of the adsorption and release assays; b) adsorption of EB by cationic MCS over a 24 h period; c) adsorption of MB by anionic MCS over a 24 h period; d) release of EB from cationic MCS in different concentrations of PBS; and e) release of MB from anionic MCS in different concentrations of PBS. Data are expressed as mean ± SD of at least three replicates.

As a straightforward method to endow MCS with light‐driven antimicrobial functionalities, photosensitizers were physically integrated into the pre‐formed porous sponge network by physical adsorption. We explore the kinetics of photosensitizer adsorption and controlled release, offering insights into dye uptake and liberation rates. Typically, the adsorption process entails multiple phases: the ingress of dye molecules from the solution to the surface of adsorbents, the diffusion of the dye molecules from the surface to the inner adsorptive sites, and the electrostatic interaction of dye molecules with active sites on the polymer's backbone. The uptake of negatively charged dye EB (1 g L^−1^) by cationic MCS was quantified, while the adsorption of positively charged dye MB (0.5 g L^−1^) by anionic MCS was studied. As illustrated in **Figure** **4**b,c, adsorption kinetics showed a rapid initial increase, leading to an equilibrium upon saturation of adsorptive sites. Cationic MCS reached adsorption equilibrium within 4 h, with an adsorption capacity of 78.15 ± 2.04 mg g^−1^, consistent with previously reported studies where highly porous cationic polyelectrolytes achieved EB adsorption capacities exceeding 100 mg g^−1^.^[^ ^41^ ^]^ Anionic MCS achieved significant MB adsorption within a 2 h immersion, reaching an equilibrium adsorption capacity of 110.36 ± 9.63 mg g^−1^, attributed to the formation of ionic complexes between MB and the COO^−^ groups in the superadsorbent network.^[^ ^42^, ^43^ ^]^ Overall, the robust adsorption of photosensitizer molecules is anticipated to be critical for the light‐activated self‐sanitizing function of MCS.

The controlled release of photosensitizers in a salt solution is another significant characteristic of synthesized cellulose sponges. In this assay, MCS impregnated with dyes—cationic MCS with EB and anionic MCS with MB—were immersed in PBS at various concentrations for up to 24 h, with the release kinetics illustrated in Figure 4d,e. A correlation between higher salt concentrations and increased dye release rates is evident for cationic and anionic MCS. In cationic MCS, EB release continued to rise over 24 h without reaching equilibrium. In contrast, anionic MCS exhibited a rapid MB release that stabilized after 1.5 h, achieving equilibrium in ≈4 h. This behavior is likely caused by competitive ionic interactions in PBS that replace dyes from binding sites on the cellulose backbone. Notably, MB crystallization was observed within ≈20 min in a 10× PBS solution, indicating reduced solubility of EB at higher PBS concentrations. In control and 0.1× PBS solutions, the MB release was minimal, attributed to strong COO‐/MB+ complex formation, as supported by previously published studies.^[^ ^44^, ^45^ ^]^ This suggests chemisorption mechanisms primarily govern MB release, with the delocalized positive charge of MB dye contributing to its strong attachment to the anionic MCS. Consequently, the ionic strength of 0.1× PBS was insufficient to rapidly disrupt MB‐MCS interactions compared to higher PBS concentrations. Overall, the unique capability for controllable adsorption and release of photosensitizers enables potential recycling and reuse of these substances in real‐world applications.

### 2.4 Cooling Properties

**Figure 5:**
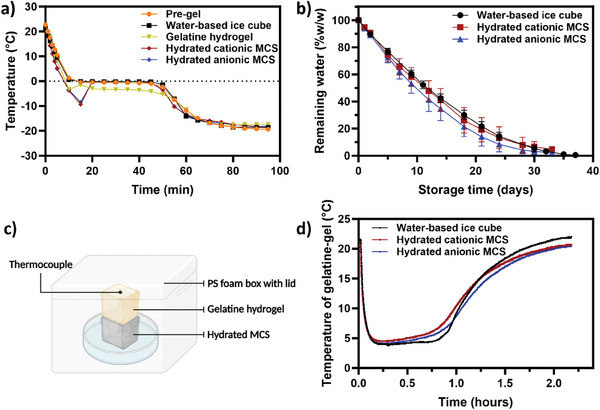
Suitability of MCS as cooling media. a) Temperature changes of subjects during the frozen process; b) water retention of hydrated MCS and water‐based ice cubes in a freezer over 40 days storage; c) schematic diagram of the cooling efficiency test device; and d) cooling curves of hydrated MCS and water‐based ice cubes. Data are expressed as mean ± SD of at least three replicates.

The cooling properties of hydrated MCS were evaluated by measuring its cooling capacity, water retention during freezing, and overall cooling efficiency. The temperature curves of water, cationic MCS, and anionic MCS during the freezing process are presented in **Figure** **5**a, along with comparisons to a non‐cross‐linked pre‐gel solution and a gelatin‐based hydrogel. The dydrated MCS demonstrated cooling capacities comparable to traditional water‐based ice, reaching ≈−19.8 °C almost simultaneously and maintaining a stable freezing point at 0 °C. Notably, the temperature profiles of both cationic and anionic MCS exhibited supercooling events, characterized by distinct kinks between 10 and 15 min. This phenomenon was absent in the pre‐gel solution. However, a slight freezing point depression to −0.5 °C was noted, attributed to free ionic species (e.g., citrate anion, ammonium action, and carboxylate anion) inhibiting water crystallization. In the case of hydrated MCS, with ionic species constrained to the porous sponge network rather than dispersed in water, the freezing points remained unaffected. Notably, gelatin‐based hydrogel displayed a less pronounced supercooling and a significant freezing point depression of 3.0 °C, potentially caused by the enhanced interactions of charged species on gelatin backbone with water molecules at a scale similar to its molecular dimension and the presence of salts.^[^ ^19^ ^]^ Conversely, initial freezing of the pre‐gel solution during the MCS fabrication produces microscopic cavities that compartmentalize water into discontinued phases, which might contribute to freezing point depression by prohibiting ice crystal formation in a homogeneous manner, but without affecting the overall freezing point.

The water retention of MCS during a 40‐day freeze at −20 °C was compared to conventional water‐based ice cubes (Figure 5b). Cationic MCS showed similar water loss rates to water‐based ice, while anionic MCS had a slightly higher rate. After a fortnight, water loss was recorded at 57.59% for water‐based ice cubes, 58.81% for cationic MCS, and 64.18% for anionic MCS. By day 33, both types of MCS had stabilized in mass, indicating complete water evaporation. Ultimately, cationic MCS retained 4.82% of their initial weight, anionic MCS 2.42%, and water‐based ice evaporated completely.

The cooling efficiency was evaluated in a real‐world simulation using a Styrofoam box as an insulating chamber that minimizes mass and heat exchange with the surroundings (Figure 5c). Within this chamber, a gelatin hydrogel cross‐linked with glutaraldehyde was used to simulate chilled food, and was positioned at the top of either a frozen hydrated MCS or a traditional water‐based ice cube. According to Figure 5d, both cationic and anionic MCS displayed similar cooling efficiencies to conventional ice. It took ≈20 min for the gelatin hydrogels to cool from 22 to 4 °C. The lowest temperatures reached were 3.8 °C with traditional ice, 4.4 °C with cationic MCS, and 4.1 °C with anionic MCS. The temperatures then remained stable for ≈30 min before gradually increasing. Notably, gelatin hydrogels cooled with traditional ice warmed up more quickly than those cooled with MCS, attributed to the faster melting of conventional ice. In summary, these findings highlight the efficacy of MCS as viable alternatives to traditional ice, demonstrating comparable cooling behavior and capacities with enhanced stability, offering potential applications in various cooling and preservation scenarios.

### 2.5 Mechanical and Thermal Stability After Multiple Freeze‐Thaw Cycles

We evaluated the reusability of MCS by examining its mechanical and thermal attributes, as well as microstructural stability, across multiple freeze‐thaw cycles (FTCs). Each cycle consisted of a 16 h freeze at −20 °C followed by an 8 h thaw at room temperature. Remarkably, the MCS retained its performances after up to 10 FTCs.

**Figure 6:**
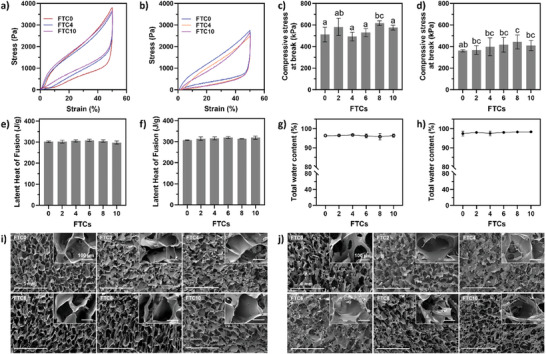
Mechanical stability over multiple FTCs.The stress–strain curves of MCS after 10 FTCs: a) cationic MCS and b) anionic MCS; changes in compressive stress at break (kPa) of MCS through 10 FTCs: c) cationic MCS and d) anionic MCS; Changes in latent heat of fusion (J/g) of hydrated MCS after up to 10 FTCs: e) cationic MCS and f) anionic MCS; changes in total water holding capacity of MCS over 10 FTCs: g) cationic MCS and h) anionic MCS; representative SEM images: i) cationic MCS and j) anionic MCS. Data are expressed as mean ± SD of at least three replicates. Means with different letters on the bars (a – c) represent significant difference (*p* < 0.05).

The stress‐strain curves for both cationic and anionic MCS post various FTCs, presented in **Figure** **6**a,b, describe the compression (50% strain) and recovery processes. The large hysteresis loop formed by the compressing and relaxing curves indicates energy dissipation within the sponge matrix through complex internal rearrangements, necessitating an extended period of shape recovery.^[^ ^46^ ^]^ Additionally, a sponge that requires more stress to reach 50% strain is considered harder. The data from Figure 6a,b show that the hardness of MCS remained stable after 10 FTCs. This mechanical durability is further supported by the maximum compressive pressure at break, as shown in Figure 6c,d, demonstrating no significant disparity between freshly prepared MCS and those subjected to 10 FTCs. These findings highlight the mechanical resilience of the proposed MCS, underlining the suitability for repeated use.

The changes in the latent heat of fusion for hydrated MCS after repeated FTCs were assessed using differential scanning calorimetry (DSC). The heat of fusion ≈0 °C, corresponding to the ice‐water transition, was measured to determine the capacity of hydrated MCS to absorb heat, a critical factor in maintaining food temperatures below hazardous levels. Results in Figure 6e,f illustrate that the average latent heat of fusion for freshly prepared cationic MCS was 308.6 J g^−1^, remaining relatively stable through 10 FTCs. Similar stability was observed in anionic MCS, starting with a latent heat of fusion of 319.4 J g^−1^ and showing no significant changes after 10 FTCs. Thermogravimetric analysis (TGA) was used to examine potential water content variations in the hydrated MCS. The results in Figure 6g,h were consistent with the earlier discussions on the changes in latent heat of fusion, confirming that no substantial differences were detected after 10 FTCs. These findings indicate a marked enhancement over previously reported gelatine‐based hydrogels, which experienced reductions in mechanical and thermal stability after five freeze‐thaw cycles (FTCs).^[^ ^18^, ^19^ ^]^

Microstructural changes following multiple FTCs were analyzed using scanning electron microscopy (SEM), as shown in Figure 6i,j. Cationic MCS largely preserved their microstructure and pore size after 10 FTCs, while anionic MCS exhibited filamentous structures and a noticeable collapse in structure after 10 cycles.

In summary, the synthesized cationic and anionic MCS exhibited robust mechanical and thermal stability, with the cationic MCS showing particular resilience in maintaining their microstructure. These findings collectively suggest that the MCS is a promising alternative to traditional ice cubes for maintaining critical temperatures, offering additional benefits in reusability and structural robustness.

### 2.6 LED Daylight‐Driven Antimicrobial Activity

**Figure 7:**
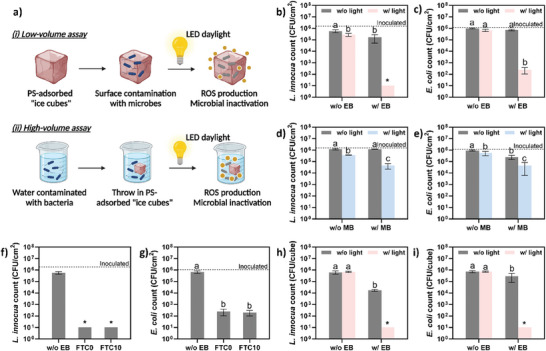
Light‐activated antimicrobial activities of MCS adsorbed with photosensitizers. a) Schematic diagrams of low‐volume and high‐volume assays. b) Inactivation of *L. innocua* on the surface of cationic MCS. c) Inactivation of *E. coli* on the surface of cationic MCS. d) Inactivation of *L. innocua* on the surface of anionic MCS. e) Inactivation of *E. coli* on the surface of anionic MCS. f) Inactivation of *L. innocua* on the surface of cationic MCS after 10 FTCs. g) Inactivation of *E. coli* on the surface of cationic MCS after 10 FTCs. h) Inactivation of *L. innocua* in high‐volume bacterial suspension. i) Inactivation of *E. coli* in high‐volume bacterial suspension. Data are expressed as mean ± SD of at least three replicates. Means with different letters on the bars represent significant difference (*p* < 0.05).

The self‐sanitizing property is crucial for reusable coolant materials, ensuring microbial food safety by reducing the risk of cross‐contamination. In this study, adsorbed photosensitizers were able to generate reactive oxygen species (ROS) upon LED daylight exposure, effectively killing microorganisms on or near the ice cube surface. The light‐driven antimicrobial activity was demonstrated in two scenarios: low‐volume and high‐volume assays. The low‐volume assay, simulating the surface cleaning of MCS‐based ice cubes, is illustrated in **Figure** **7**a. Gram‐positive *L. innocua* and Gram‐negative *E. coli*, representing foodborne pathogens, were inoculated on the ice cube surfaces, resulting in levels of 1.8 × 10^6^ and 1.1 × 10^6^ CFU cube^−1^, respectively. As shown in Figure 7b,c, EB‐adsorbed cationic MCS, after 1 h of LED daylight irradiation, substantially reduced bacterial counts of about a 5‐log reduction for *L. innocua* and a 4‐log reduction for *E. coli*. In contrast, the EB‐adsorbed MCS without light irradiation showed less than 1‐log reduction for both bacteria and control groups without EB showed no significant bacterial reduction, regardless of light exposure. In contrast, MB‐adsorbed anionic MCS demonstrated lower antimicrobial activity, with less than 2‐log reductions for both bacteria after 1 h of daylight exposure, as shown in Figure 7d,e. This reduced effect might be due to the aggregation of MB when adsorbed onto MCS, limiting ROS production.^[^ ^47^ ^]^ This aggregation‐induced decrease in MB effectiveness was observed in a study using MB to inactivate *Candida albicans*.^[^ ^48^ ^]^ The antimicrobial efficacy of EB‐adsorbed cationic MCS remained robust after 10 FTCs. Figure 7f,g shows no significant activity loss compared to the freshly prepared MCS, inactivating ≈5‐log CFU cm^−2^ of *L. innocua* and 4‐log CFU cm^−2^ of *E. coli* after 1 h of LED daylight exposure. These results are consistent with previous observations that multiple FTCs did not compromise the MCS's mechanical, thermal stability, or microstructure (Figure 6). The high‐volume assay further confirmed the robust antimicrobial capability of EB‐adsorbed cationic MCS, achieving a 5‐log reduction in both *L. innocua* and *E. coli* when immersed in a 1 mL bacterial suspension at a concentration of ≈6‐log CFU mL^−1^ and exposed to LED daylight for 1 h. In the absence of EB, the MCS were ineffective in killing bacteria. Notably, EB‐adsorbed cationic MCS in the dark still led to ≈1.5‐log reduction for *L. innocua* and 0.5‐log reduction for *E. coli*, suggesting some antimicrobial activity from the released EB.

These findings highlight the potential of EB‐adsorbed cationic MCS as an effective antimicrobial solution in reusable cooling applications, presenting a promising approach to enhancing food safety in the cold chain.

### 2.7 Prevention of Cross‐Contamination of Fresh Produce and Seafood

**Figure 8:**
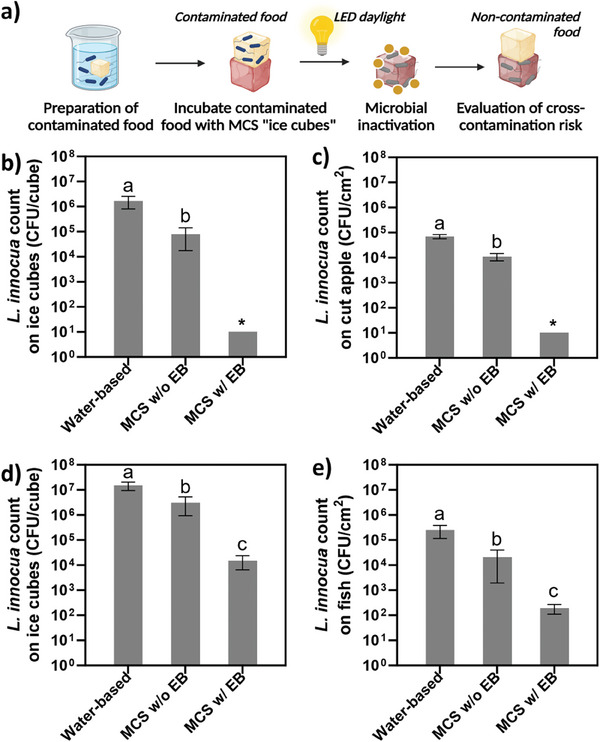
Effect of MCS on the prevention of food cross‐contamination using model food systems. a) Schematic diagram of cross‐contamination assay. Using cut apple as a model food: b) bacterial count on the ice cubes after contact with contaminated apple cubes and c) bacterial count transferred from contaminated ice cubes to non‐contaminated apple samples. Using salmon fillets as model food: d) bacterial count on the ice cubes after contact with contaminated salmon fillets and e) bacterial count transferred from contaminated ice cubes to non‐contaminated salmon fillets. Data are expressed as mean ± SD of at least three replicates. Means with different letters on the bars (a – c) represent significant difference (*p* < 0.05).

Building on the demonstrated antimicrobial efficacy of EB‐adsorbed cationic MCS, we further exploit their ability to prevent cross‐contamination in the food cold chain, a crucial factor in reducing foodborne disease outbreaks. We designed assays to evaluate the EB‐adsorbed cationic MCS to prevent bacterial transfer between food items, using fresh‐cut apples and salmon fillets as models for produce and seafood. Rifampicin‐resistant *L. innocua* was used to represent the foodborne pathogen while screening out the influences from natural flora. The results in **Figure** **8** demonstrate that EB‐adsorbed cationic MCS significantly reduced cross‐contamination between food samples. Traditional ice facilitated the transfer of 4.8‐log CFU cm^−2^ of bacteria to uncontaminated apples. In contrast, following one hour of LED daylight exposure, EB‐adsorbed MCS completely inactivated the bacteria on the surface, thereby preventing any bacterial transfer to the non‐contaminated apples. Without EB, the cationic MCS did not inhibit microbial cross‐contamination, with 4.0 log CFU cm^−2^ transferring to apple surfaces. For salmon fillets, which presented a greater challenge due to higher organic content, the EB‐adsorbed MCS resulted in a markedly lower bacterial transfer of 2.2 log CFU cm^−2^, significantly less than the 5.4 log CFU cm^−2^ observed with traditional ice cubes. These results demonstrate the potential of EB‐adsorbed MCS in substantially reducing the risk of microbial transfer in the cold chain.

In addition to the microbial cross‐contamination assay, an additional experiment was performed to assess the transfer of photosensitizers from ice cubes into a simulated food material, specifically PVA gels. The results in Figure S8 (Supporting Information) indicate that the MCS‐based ice cubes transferred a minimal amount of photosensitizers to the simulated food matrices, in contrast to the results observed when photosensitizers were directly incorporated into conventional water‐based ice cubes. After a 1‐hour period of contact between the ice cubes and PVA gels, the traditional ice cubes had completely melted, leading to significant contamination of the PVA gels with photosensitizers. Conversely, the MCS‐based ice cubes demonstrated exceptional water retention capabilities, releasing only trace amounts of photosensitizers to the model food material, quantified as less than 0.77 µg per gel. This combination of potent daylight‐induced antimicrobial activity with minimal food contamination presents a viable alternative to traditional ice cubes in the cold chain, thereby contributing to improved food safety and quality.

## 3 Conclusion

This study highlights the practicality of microporous cellulose sponges (MCS) as a sustainable substitute for traditional water‐based ice cubes in various cooling applications. The cationic MCS, when adsorbed with EB, exhibits potent self‐sanitizing properties. It effectively inactivates over 5 log CFU cm^−2^ of *L. innocua* and 4 log CFU cm^−2^ of *E. coli* after an hour of exposure to green LED daylight. This functionality positions the MCS as an effective tool in mitigating microbial cross‐contamination, proving especially beneficial in transporting and storing perishable foods such as fresh produce and seafood. The adsorption and release rates of the photosensitizers in MCS can be modulated by varying the salt solution concentrations. This adaptability enhances the reusability of MCS, enabling them to function as self‐sanitizing “ice cubes”. Notably, the proposed hydrated MCS parallels conventional ice regarding cooling behavior and heat adsorption capabilities. It also demonstrate robust mechanical and thermal stability, retaining their structural integrity even after multiple freeze‐thaw cycles. The high compressibility and ability to regain shape after compression makes them space‐efficient and cost‐effective for transit and storage. Despite these promising results, certain challenges remain. For instance, the bacterial inactivation efficacy of anionic MCS with MB is less pronounced and warrants further exploration. Overall, this study lays the groundwork for future research and development in sustainable cooling technologies.

## 4 Experimental Section

### Chemicals and Reagents

Sodium carboxymethyl cellulose (NaCMC, MW 700 kDa), quaternized hydroxyethyl cellulose ethoxylate (QHEC, MW 720 kDa), gelatine from bovine skin, poly(vinyl alcohol), glutaric dialdehyde 50 wt.% solution in water, tryptic soy broth (TSB), and tryptic soy agar (TSA) were purchased from Sigma‐Aldrich (Auckland, New Zealand). Methylene blue was purchased from BHD Chemicals Ltd (Auckland, New Zealand), and erythrosine B was purchased from AK Science (Union City, CA, USA). Citric acid (CA), phosphate Buffered Saline (PBS, 10× solution), sodium hydroxide, and hydrochloric acid were purchased from Fisher Scientific (Pittsburgh, PA, USA).

### Preparation of MCS)

Cationic and anionic MCS were synthesized by cross‐linking cellulose with citric acid, following a modified version of a previously reported method.^[^ ^49^ ^]^ It was aimed to create cellulose sponges with varied mechanical and thermal properties, as well as differing net surface charges. To achieve this, different weight ratios of NaCMC to QHEC were utilized. The specific NaCMC:QHEC weight ratios explored were 3:1, 2:1, 1:1, 1:2, and 1:3. The total polymer solid concentration was adjusted to a range of 2%−4% for the cationic MCS and 0.5%−2% for the anionic MCS. For both types of MCS, CA concentrations varied from 5% to 20% w/w relative to the polymer weight.

To prepare the pre‐gel solution, NaCMC was added to the CA solution and stirred at 200 rpm at room temperature until a clear solution was achieved. Subsequently, QHEC was added to this mixture and continuously stirred at room temperature for 30 min, or until the solution transitioned from clear to uniformly cloudy. This pre‐gel solution was then subjected to reduced pressure (≈0.3 bar) to eliminate entrapped air bubbles, which could otherwise lead to large pores in the final MCS. Subsequently, the pre‐gel solutions were transferred to a mold and successively froze at −20 °C for 24 h and −80 °C for 24 h, followed by lyophilization (freeze drying) for 48 h to remove water. Post‐freezing, the pre‐gel samples underwent cross‐linking in an oven set to 80 °C overnight. The resulting cellulose sponges were then preserved in a glass vacuum desiccator until further use.

### Characterization of MCS

*Surface Chemistry*: The surface chemistry of the synthesized materials was characterized using dynamic light scattering (DLS) and Attenuated Total Reflection‐Fourier Transform Infrared (ATR‐FTIR) spectroscopy. The net surface charge (ζ‐potential) of the pre‐gel under different mixing ratios were assessed using the Nano‐ZS zeta sizer (Malvern Instruments, Worcestershire, UK). The change in surface chemical groups before and after cross‐linking was monitored by ATR‐FTIR spectroscopy (Bruker Vertex 70, Bruker, Billerica, MA, USA).

*Equilibrium Water Adsorption Ratio*: The equilibrium water adsorption ratio of the optimized cationic and anionic MCS was quantitatively assessed by immersing the synthesized MCS in distilled water for predetermined periods. The initial dry mass of each MCS cube was recorded before immersion. Subsequently, the wet mass of the fully hydrated MCS was determined at intervals of 1, 2, and 24 h during the immersion period. Photographic documentation of the MCS was conducted before and after a 24 h soaking period. The water adsorption ratio was calculated using the following equation:

(1) $$AR\left(\%\right)\;=\frac{M_{W}-M_{D}}{M_{D}}\;\times 100$$

where *AR* (%) is the adsorption ratio, *M_W_* (mg) is the wet mass of the hydrated MCS, and *M_D_* (mg) is the dry mass of the MCS.

*Dehydration and Rehydration Cycles*: The water adsorption capacity of the synthesized MCS was evaluated over multiple dehydration and rehydration cycles. Cycle 0 refers to the synthesized MCS after lyophilization and cross‐linking at 80 °C. Initially, the MCS was left to hydrate in distilled water overnight. The weight of the dry MCS and that of the hydrated MCS were recorded. Subsequently, the hydrated MCS was placed in an oven at 30 °C for 24 h to facilitate water evaporation, and the weight of the dried MCS was recorded. This alternating hydration and dehydration process was repeated ten times for both the cationic and anionic MCS to evaluate their water retention and release capacities effectively.

*Compression Resistance*: The mechanical stability of the synthesized MCS was evaluated using a texture analyzer (TA‐XT Plus, Stable Micro Systems, Godalming, UK). The integrity of the MCS was tested in compression mode, employing a cylindrical probe advancing at a rate of 0.60 mm s^−1^. The compression depth of the probe was set to 9 mm, with an applied trigger force of 0.001 N. The maximum pressure exerted at the point of fracture (kPa) was recorded and averaged to determine the compression resistance of MCS.

*Cooling Behaviors*: Cooling capacity was measured by incubating the samples at −20 °C. The performance of the hydrated MCS was evaluated in comparison to water ice, a non‐cross‐linked pre‐gel solution, and a gelatin‐based food sample. All the samples started at ambient room temperature. The temperature variations of each sample were tracked using a multi‐channel handheld thermocouple temperature data logger (model S220‐T8, manufactured by Huato in Shenzhen, China).

The cooling efficiency of hydrated MCS and water‐based ice cubes was tested using a custom‐designed device, as shown in Figure 5c, to simulate a practical food cooling scenario. A gelatin‐based hydrogel (1 × 1 × 1 cm^3^), representing the food sample, was prepared by cross‐linking 10% w/w gelatin with 1% w/w 50% glutaraldehyde solution.^[^ ^50^ ^]^ Prior to the test, the MCS were hydrated and then frozen at −20 °C overnight, with water‐based ice cubes used as the control. To assemble the testing device, each frozen hydrated MCS or water‐based ice cube was placed inside a polystyrene foam box, with the gelatin‐based food sample at room temperature placed atop the ice cube. The foam box was then kept at room temperature for 2 h, during which the internal temperature of the gelatin‐based food samples was monitored using a multi‐channel handheld thermocouple temperature data logger (model S220‐T8, Huato, Shenzhen, China).

*Recovery of MCS after Vacuum Storage*: The hydrated MCS was subjected to full compression and vacuum‐sealed in a bag using a vacuum sealer (Wedderburn, Auckland, New Zealand). These vacuum‐sealed samples were then stored at ambient room temperature. Following one week of storage, the samples were rehydrated using distilled water. The height of the rehydrated MCS was measured and compared to their original height.

### Adsorption and Release of Photosensitizers

To assess the dye uptake efficiency of both cationic and anionic MCS, methylene blue (MB), a cationic dye, and erythrosine B (EB), an anionic dye were employed, as model photosensitizers. Initially, the MCS were weighed in their dehydrated state, and the measurements were recorded. For the adsorption tests, cationic MCS were immersed in 1 g L^−1^ EB solution, using a volume of 5 mL, while anionic MCS were placed in a 5 mL MB solution with a concentration of 0.5 g L^−1^. After allowing them to incubate at ambient temperature for a predetermined period, the residual dye concentration in each solution was quantified using a UV/Vis spectrophotometer (model UV‐1280, Shimadzu, Kyoto, Japan) at a predetermined wavelength of 526 and 664 nm for EB and MB, respectively. To quantify the dye adsorption capacities of the MCS, the following formula was applied:

(2) $$q_{e}=\frac{\left(c_{0}-c_{e}\right)V}{M_{D}}\;$$

where *q_e_* (mg g^−1^) is the concentration of dye adsorbed by the MCS, *c_0_* (mg L^−1^) is the initial concentration of the dye solution, *c_e_* is the final or equilibrium concentration of the dye solution, *V* (L) is the volume of the dye solution (L), and *M_D_* (g) is the dry mass of the MCS.

The kinetics of photosensitizer release from MCS into surrounding solutions were investigated. Initially, the cationic and anionic MCS were preconditioned by immersing them overnight in solutions of EB and MB, respectively. This incubation allowed for sufficient photosensitizer adsorption by the MCS. After this period, the MCS samples were thoroughly rinsed with distilled water to remove any photosensitizers that had not been adsorbed. Following the rinsing process, the MCS samples were placed into phosphate‐buffered saline (PBS) solutions of varying concentrations: 10×, 1×, and 0.1×. The release of photosensitizers from the MCS into the saline was monitored using a UV/Vis spectrophotometer, with measurements of absorbance taken at desired time intervals over a period of up to 48 h.

Another assay aimed to evaluate the release of adsorbed photosensitizers from MCS to simulated food materials. For this purpose, polyvinyl alcohol (PVA) gels were prepared using a method adapted from a previous study,^[^ ^51^ ^]^ serving as a model food product. Both EB‐adsorbed cationic MCS and MB‐adsorbed anionic MCS were prepared according to the procedures outlined in Section “Adsorption and release of photosensitizers”. As a control, water‐based ice cubes containing either a 150 µg mL^−1^ EB solution or a 300 µg mL^−1^ MB solution were frozen to create EB and MB ice cubes, respectively. These ice cubes were then placed atop the PVA gels and left to stand for 1 h at room temperature. Subsequently, the transfer of photosensitizers from the ice cubes to the PVA gels was quantified using the previously described method.

### Effect of Repeated Freeze‐Thaw Cycles (FTCs)

The effects of repeated FTCs on the properties of MCS samples were characterized by analyzing the mechanical properties, structural integrity, latent heat of fusion, and moisture content. The process began with the MCS being soaked in distilled water at ambient temperature for an overnight period. Subsequently, they underwent a freezing stage at −20 °C lasting 16 h, followed by a thawing stage at room temperature for 8 h. This cycle was repeated for 2, 4, 6, 8, and 10 iterations, with samples collected after each specified number of cycles for various analysis.

To evaluate the mechanical properties of MCS, a single‐column load frame (Instron model 5943, Massachusetts, USA) was employed. The samples prepared for this test measured 1.5 cm in diameter and 1 cm in height. For microstructural analysis, field emission scanning electron microscopy (FE‐SEM, Hitachi Ltd., model SU‐70, Tokyo, Japan) was used. Before SEM examination, the MCS samples were freeze‐dried to remove all moisture and then coated with platinum for 40 s in an argon atmosphere to render them conductive for imaging. Latent heat measurements of the hydrated MCS after various FTCs were conducted using differential scanning calorimetry (DSC‐60, Shimadzu Corporation, Pleasanton, CA). The procedure involved tracking the heat absorption and release of hydrated MCS samples from −30 to 10 °C at a rate of 1 °C min^−1^, under a protective nitrogen atmosphere at 50 mL min^−1^. The calculation of the latent heat of fusion was performed using a similar method as previously reported,^[^ ^18^ ^]^ using the latent heat of fusion of water ice (334.5 J g^−1^) as a reference. Furthermore, the moisture content was analyzed via thermogravimetric analysis (TGA, TA Instruments, SDT‐Q600, New Castle, DE), where the first derivative of the TGA curve was instrumental in identifying water loss.

### Antimicrobial Assays

The antimicrobial efficacy of the photosensitizer‐adsorbed MCS was evaluated against both Gram‐negative *Escherichia coli* (ATCC 25 922) and Gram‐positive *Listeria innocua* (ATCC 33 090) to assess the bactericidal properties. A bacterial culture was obtained by inoculating a single colony into 10 mL of tryptic soy broth (TSB) and incubating it at 37 °C with agitation at 250 rpm for 16 h. Post‐incubation, the culture underwent centrifugation at 3000 ×g and was washed twice with sterile PBS to achieve a final bacterial concentration of ≈1 × 109 CFU mL^−1^.

Prior to the antimicrobial test, the MCS samples were soaked overnight in EB (1 g L^−1^) or MB (0.5 g L^−1^) solutions, followed by rinsing with sterile distilled water to remove excess dye. For the control group, the MCS samples were soaked in sterile distilled water. Excess surface water was carefully removed, and the MCS samples were placed in 24‐well plates and frozen at −20 °C for up to 4 h.

The in vitro antimicrobial efficacy of MCS was evaluated under both low‐volume and high‐volume conditions. In the low‐volume scenario, 100 µL of bacterial suspension (10^7^ CFU mL^−1^) was applied on top of each MCS cube. In the high‐volume scenario, 1 mL of bacterial suspension (10^6^ CFU mL^−1^) was added to each well containing a MCS cube. The samples were placed in an ice bath and then exposed to LED light irradiation in a light chamber, with cationic MCS (EB‐adsorbed) under green LED light (540 nm, ≈3.5 mW cm^−2^) and anionic MCS (MB‐adsorbed) under red LED light (630 nm, ≈3.5 mW cm^−2^). Control samples were kept in the dark for the same duration. After irradiation, an additional 1 mL of sterile PBS was added to each well for bacterial recovery, followed by serial dilution and microbial plate counting.

To evaluate the capability of preventing microbial cross‐contamination between food and MCS‐based ice cubes, the self‐sanitizing activity of photosensitizer‐adsorbed MCS was assessed on fresh cut apples and salmon fillets (both diced into 1 × 1 × 1 cm^3^ cubes), inoculated with 300 µL of a 1 × 10^8^ CFU mL^−1^ rifampicin resistant *L. innocua* suspension. After an hour for bacterial adhesion, the contaminated food samples were placed on the MCS cubes for 30 min. After treatment, the MCS cubes were illuminated with LED light for 60 min, while control groups were kept in darkness. Bacteria were recovered using sterile PBS, serially diluted, and plated on TSA with 50 mg L^−1^ rifampicin for enumeration.

### Statistical Analysis

Statistical analysis was conducted using GraphPad Prism software V.10.1.0 (GraphPad Software, Inc., La Jolla, CA). Significant differences between treatments were identified using one‐way ANOVA followed by Tukey's pairwise comparisons, maintaining a 95% confidence interval.

## Conflict of Interest

The authors have no conflicts of interest.

## Author Contributions

Y.L., B.X., and Y.L. contributed equally to this work. Y.L. performed methodology, data collection and analysis, visualization, wrote the original draft. B.X. performed conceptualization, methodology, wrote, reviewed, and edited the draft. Y.L. performed methodology, data collection and analysis, visualization, wrote the original draft. S.‐Y.Q. performed wrote, reviewed, and edited the draft, supervision. K.H. performed conceptualization, data collection and analysis, wrote, reviewed, and edited the draft, supervision.

## Supporting information

Supporting Information

## Data Availability

The data that support the findings of this study are available from the corresponding author upon reasonable request.

## References

[1] L. Settanni , R. Gaglio , C. Stucchi , S. De Martino , N. Francesca , G. Moschetti , Ann. Microbiol. 2017, 67, 827. 10.1111/jam.13624 29080227

[2] C. L. Baylis , in Enterobacteriaceae, (Ed: C. D. W. Blackburn ), Woodhead Publishing, Cambridge 2006, pp. 624.

[3] S. Don , K. A. M. Xavier , S. T. Devi , B. B. Nayak , N. Kannuchamy , LWT 2018, 97, 295.

[4] D. I. Onwude , G. Chen , N. Eke‐emezie , A. Kabutey , A. Y. Khaled , B. Sturm , Processes 2020, 8, 1431.

[5] L. Feliciano , J. Lee , J. A. Lopes , M. A. Pascall , J. Food Sci. 2010, 75, M231. 20546415 10.1111/j.1750-3841.2010.01583.x

[6] H. Waite , D. Gramaje , M. Whitelaw‐Weckert , P. Torley , W. J. Hardie , Phytopathol. Mediterr. 2013, 52, 359.

[7] J. Gao , H. Jang , L. Huang , K. R. Matthews , Int. J. Food Microbiol. 2020, 323, 108593 32224348 10.1016/j.ijfoodmicro.2020.108593

[8] R. A. Stein , M. Chirilã , in Foodborne Diseases, 3rd ed., (Eds: C. E. R. Dodd , T. Aldsworth , R. A. Stein , D. O. Cliver , H. P. Riemann ), Academic Press, Cambridge 2017, pp. 65–103.

[9] J.‐H. Shin , S. Chang , D.‐H. Kang , J. Appl. Microbiol. 2004, 97, 916. 15479406 10.1111/j.1365-2672.2004.02343.x

[10] S. Koseki , K. Fujiwara , K. Itoh , J. Food Prot. 2002, 65, 411. 11848576 10.4315/0362-028x-65.2.411

[11] J. Zhou , H. Zhao , G. Xue , Huanjing Kexue 2003, 24, 45. 12916201

[12] J. Fawell , Food Chem. Toxicol. 2000, 38, S91. 10.1016/s0278-6915(99)00129-5 10717377

[13] M. Usman , M. Hüben , S. Hahn , S. Wieck , A. Kehrer‐Berger , V. Linnemann , T. Wintgens , Environ. Sci. Eur. 2023, 35, 77.

[14] N. Parveen , S. Chowdhury , S. Goel , Environ. Sci. Pollut. Res. 2022, 29, 85742. 10.1007/s11356-021-18316-2 PMC8799444 35091954

[15] T. Lin , J. J. Wang , J. B. Li , C. Liao , Y. J. Pan , Y. Zhao , J. Agric. Food Chem. 2013, 61, 8695. 23947475 10.1021/jf4019933

[16] W. Lan , B. Zhang , L. Liu , T. Pu , Y. Zhou , J. Xie , J. Sci. Food Agric. 2023, 103, 3787. 36224103 10.1002/jsfa.12269

[17] H. Hampikyan , E. B. Bingol , O. Cetin , H. Colak , J Water Health 2017, 15, 410. 28598345 10.2166/wh.2017.159

[18] J. Zou , L. Wang , G. Sun , ACS Sustainable Chem. Eng. 2021, 9, 15365.

[19] J. Zou , L. Wang , G. Sun , ACS Sustainable Chem. Eng. 2021, 9, 15357.

[20] J. Zou , L. Wang , G. Sun , ACS Appl. Mater. Interfaces 2023, 15, 34087. 37428710 10.1021/acsami.3c06658

[21] J. Zou , A. O. Sbodio , B. Blanco‐Ulate , L. Wang , G. Sun , Adv. Funct. Mater. 2022, 32, 2201347.

[22] B. Rismanchi , R. Saidur , G. BoroumandJazi , S. E. Ahmed , Renewable Sustainable Energy Rev. 2012, 16, 5741.

[23] S. D. M. Nair , K. H. Lau , Int. Soc. Hortic. Sci. 2013, 1012, 1311.

[24] Y. Qiu , K. Park , Adv. Drug Delivery Rev. 2012, 64, 49. 10.1016/s0169-409x(01)00203-4 11744175

[25] A. T. Paulino , M. R. Guilherme , A. V. Reis , G. M. Campese , E. C. Muniz , J. Nozaki , J. Colloid Interface Sci. 2006, 301, 55. 16740270 10.1016/j.jcis.2006.04.036

[26] G. Li , Y. Du , Y. Tao , H. Deng , X. Luo , J. Yang , Carbohydr. Polym. 2010, 82, 706.

[27] R. Juang , R. Shiau , J. Membr. Sci. 2000, 165, 159.

[28] K. J. De France , T. Hoare , E. D. Cranston , Chem. Mater. 2017, 29, 4609.

[29] D. Zhao , J. Huang , Y. Zhong , K. Li , L. Zhang , J. Cai , Adv. Funct. Mater. 2016, 26, 6279.

[30] S. Wang , L. Yu , S. Wang , L. Zhang , L. Chen , X. Xu , Z. Song , H. Liu , C. Chen , Nat. Commun. 2022, 13, 3408. 35729107 10.1038/s41467-022-30224-8 PMC9213515

[31] B. Fei , R. A. Wach , H. Mitomo , F. Yoshii , T. Kume , J. Appl. Polym. Sci. 2000, 78, 278.

[32] N. S. V. Capanema , A. A. P. Mansur , S. M. Carvalho , L. L. Mansur , C. P. Ramos , A. P. Lage , H. S. Mansur , J. Appl. Polym. Sci. 2018, 135, 45812.

[33] J. Kang , S. I. Yun , Gels 2022, 9, 20.36661788

[34] A. Kausar , S. T. Zohra , S. Ijaz , M. Iqbal , J. Iqbal , I. Bibi , S. Nouren , N. El Messaoudi , A. Nazir , Int. J. Biol. Macromol. 2023, 224, 1337. 36309237 10.1016/j.ijbiomac.2022.10.220

[35] J. C. Roy , A. Ferri , S. Giraud , G. Jinping , F. Salaün , Int. J. Mol. Sci. 2018, 19, 2521. 30149641 10.3390/ijms19092521 PMC6163483

[36] F. Wu , Y. Pang , J. Liu , Nat. Commun. 2020, 11, 4502. 32908136 10.1038/s41467-020-18308-9 PMC7481780

[37] W. Feng , Z. Wang , Adv. Sci. 2023, 10, 2303326. 10.1002/advs.202303326 PMC10558674 37544909

[38] Q. Lv , M. Wu , Y. Shen , Colloids Surf., A 2019, 583, 123972.

[39] M. Shan , C. Liu , L. Shi , L. Zhang , Y. Lin , S. Zhang , Z. Zhu , X. Wang , X. I. Zhuang , Polymers 2019, 11, 1281. 31374838 10.3390/polym11081281 PMC6723784

[40] M. K. Joshi , H. R. Pant , A. P. Tiwari , B. Maharjan , N. Liao , H. J. kim , C. H. Park , C. S. Kim , Carbohydr. Polym. 2016, 136, 154. 26572341 10.1016/j.carbpol.2015.09.018

[41] S. Kovačič , N. Drašinac , A. Pintar , E. Žagar , Langmuir 2018, 34, 10353. 30080054 10.1021/acs.langmuir.8b01645

[42] I. Ayouch , I. Kassem , Z. Kassab , I. Barrak , A. Barhoun , J. Jacquemin , K. Draoui , M. E. Achaby , Surf. Interfaces 2021, 24, 101124.

[43] S. S. Shah , B. Ramos , A. C. Teixeira , Water (Basel) 2022, 14, 3313.

[44] B. H. Vilsinski , P. R. Souza , A. C. de Oliveira , C. M. C. Filho , A. J. M. Valente , E. C. Muniz , O. Borges , A. P. Gerola , W. Caetano , A. F. Martins , Mater. Today Commun. 2021, 26, 101889.

[45] C. T. Cesco , A. J. M. Valente , A. T. Paulino , Pharmaceutics 2021, 13, 842. 34200364 10.3390/pharmaceutics13060842 PMC8228472

[46] K. Yu , Q. Ge , H. J. Qi , Nat. Commun. 2014, 5, 3066. 24423789 10.1038/ncomms4066

[47] B. Leung , P. Dharmaratne , W. Yan , B. C. L. Chan , C. B. S. Lau , K. Fung , M. Ip , S. S. Y. Leung , J. Photochem. Photobiol., B 2020, 203, 111776. 31931388 10.1016/j.jphotobiol.2020.111776

[48] G. A. Collina , F. Freire , T. P. C. Santos , N. G. Sobrinho , S. Aquino , R. A. Prates , D. D. F. Teixeira da Silva , A. C. R. T. Horliana , C. Pavani , Photochem. Photobiol. Sci. 2018, 17, 1355. 30183793 10.1039/c8pp00238j

[49] C. Demitri , R. Del Sole , F. Scalera , A. Sannino , G. Vasapollo , A. Maffezzoli , L. Ambrosio , L. Nicolais , J. Appl. Polym. Sci. 2008, 110, 2453.

[50] A. Oryan , A. Kamali , A. Moshiri , H. Baharvand , H. Daemi , Int. J. Biol. Macromol. 2018, 107, 678. 28919526 10.1016/j.ijbiomac.2017.08.184

[51] H. Adelnia , R. Ensandoost , S. Shebbrin Moonshi , J. N. Gavgani , E. I. Vasafi , H. T. Ta , Eur. Polym. J. 2022, 164, 110974.

