# Supplementary material for: Eco‐Friendly and Self‐Sanitizing Microporous Cellulose Sponge (MCS)‐Based Cooling Media for Mitigating Microbial Cross‐Contamination in the Food Cold Chain
Source: Adv Sci (Weinh). 2024 Mar 28;11(21):2309753. doi: 10.1002/advs.202309753 (PMC11151029; doi:10.1002/advs.202309753)
Supplement: Supplementary file 1 — Supporting Information [file ADVS-11-2309753-s001.pdf]

ADVANCED SCIENCE
Open Access

Supporting Information

for *Adv. Sci.*, DOI 10.1002/advs.202309753

Eco-Friendly and Self-Sanitizing Microporous Cellulose Sponge (MCS)-Based Cooling Media for Mitigating Microbial Cross-Contamination in the Food Cold Chain

*Yijun Liu, Boyang Xu, Yingxin Li, Siew-Young Quek and Kang Huang**

Supporting Information

Eco-Friendly and Self-Sanitizing Microporous Cellulose Sponge (MCS)-Based Cooling Media for Mitigating Microbial Cross-Contamination in the Food Cold Chain

Yijun Liu †,#, Boyang Xu †,#, Yingxin Li †,#, Siew-Young Quek †, Kang Huang ‡*

Author Affiliation

† School of Chemical Sciences, The University of Auckland, Auckland, 1142, New Zealand

‡ Department of Biological Systems Engineering, Washington State University, Pullman, WA 99164, USA

# These authors contributed equally.

Contact information for * Corresponding Author

Kang Huang, Ph.D.

Assistant Professor

Department of Biological Systems Engineering

Washington State University

PO Box 646120

Pullman, WA 99164, USA

+1 509 335 1232 (phone)

kang.huang@wsu.edu (email)

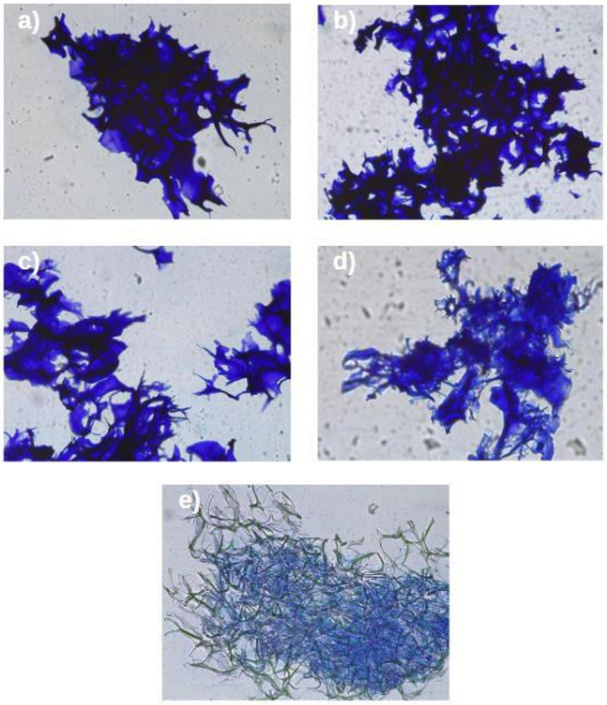

Figure S1 Homogenized MCS stained with methylene blue (MB) using different NaCMC:QHEC weight ratios: a) 3:1, b) 2:1, c) 1:1, d) 1:2, and e) 1:3.

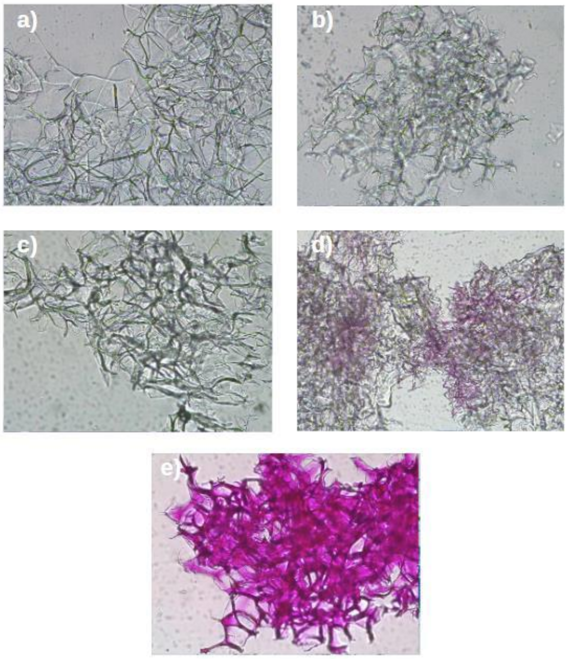

Figure S2 Homogenized MCS stained with erythrosine B (EB) using different NaCMC:QHEC weight ratios: a) 3:1, b) 2:1, c) 1:1, d) 1:2, and e) 1:3.

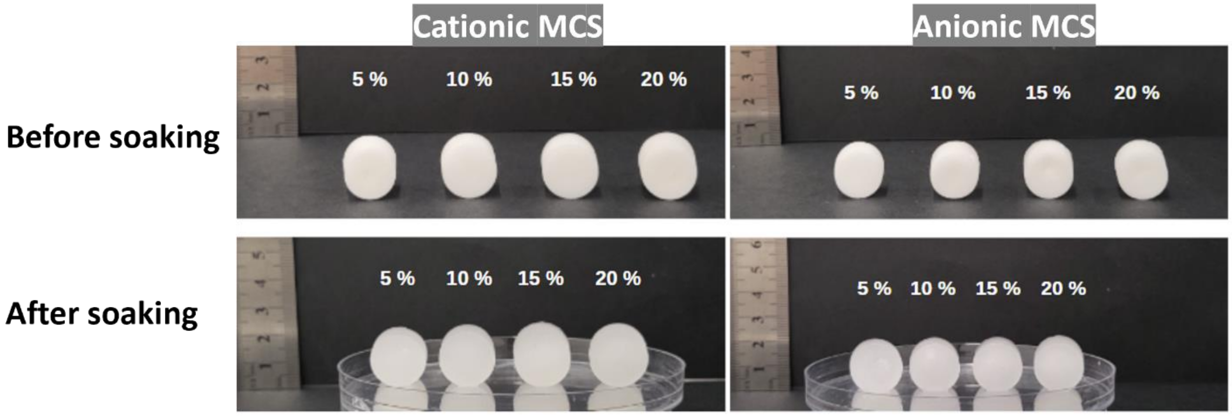

Figure S3 Representative photographs of cationic MCS (3:1, 2% solid content) and anionic MCS (3:1, 2% solid content) with different citric acid concentrations, shown before (top) and after (bottom) soaking in water for 24 hours.

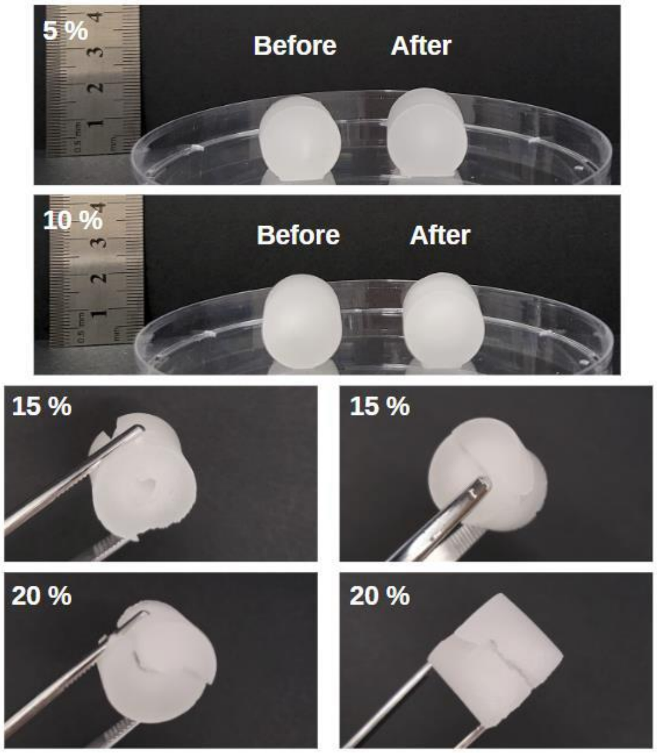

Figure S4 Representative photographs of the cationic MCS (NaCMC:QHEC = 1:3, 2% w/w solid content) in different citric acid concentrations after compression tests.

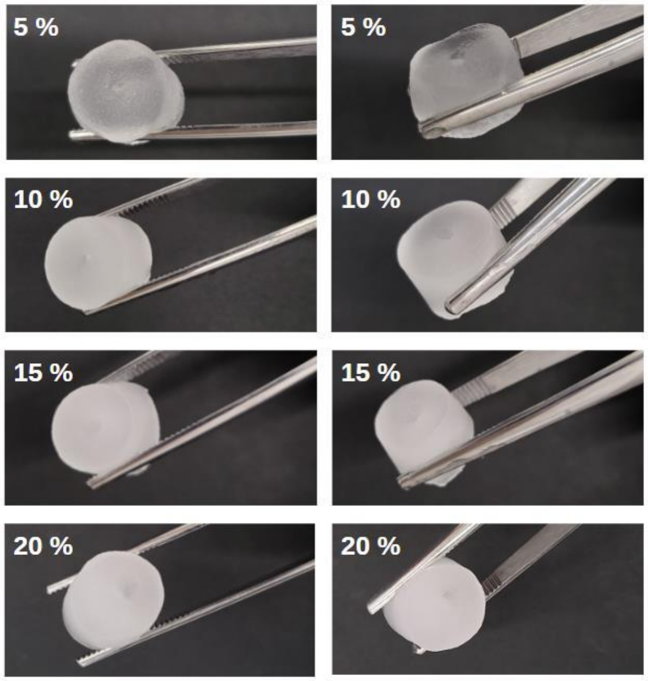

Figure S5 Representative photographs of the anionic MCS (NaCMC:QHEC = 3:1, 2% w/w solid content) in different citric acid concentrations after compression tests.

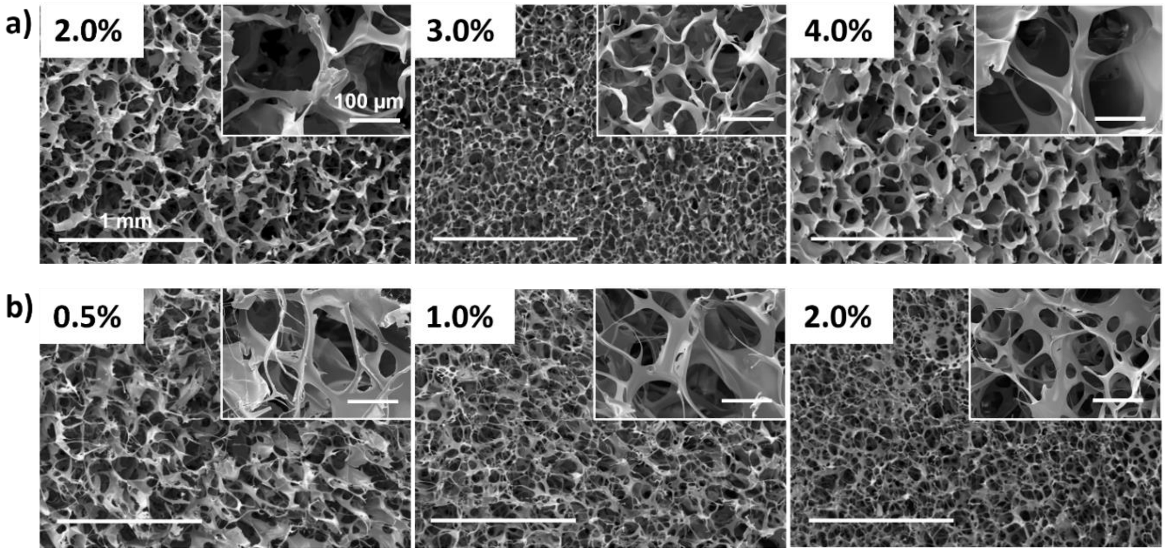

Figure S6 Representative SEM images of MCS with varying solid polymer contents and 10% w/w CA include: (a) cationic MCS with a NaCMC:QHEC weight ratio of 1:3; (b) anionic MCS with a NaCMC:QHEC weight ratio of 3:1.

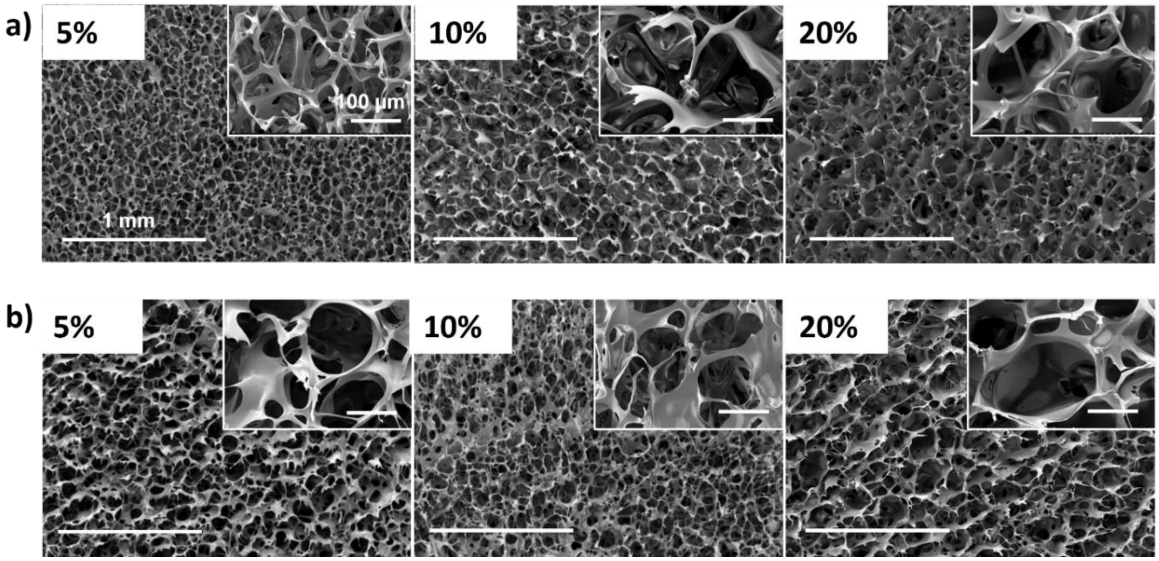

Figure S7 Representative SEM images of MCS with varying concentrations of CA include: (a) cationic MCS with a NaCMC:QHEC weight ratio of 1:3 and a total solid polymer content of 4% w/w; (b) anionic MCS with a NaCMC:QHEC weight ratio of 3:1 and a total solid polymer content of 2% w/w.

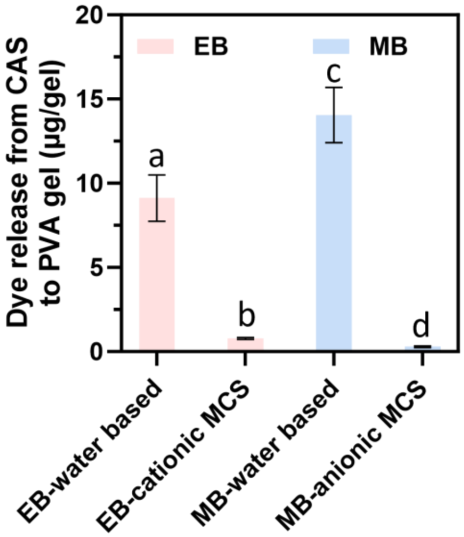

Figure S8 Release of adsorbed photosensitizers from ice cubes to simulated food materials after 1-h incubation at room temperature. Data are expressed as mean ± SD of at least three replicates. Means with different letters on the bars (a – d) represent significant difference ($p < 0.05$).
